# Classification by Nurses’ Work Values and Their Characteristics: Latent Profile Analysis of Nurses Working in Japanese Hospitals

**DOI:** 10.3390/nursrep13020077

**Published:** 2023-06-08

**Authors:** Yukari Hara, Hideyuki Hirayama, Nozomu Takada, Shoko Sugiyama, Masako Yamada, Miki Takahashi, Keita Toshi, Kyoko Asakura

**Affiliations:** 1 Graduate School of Medicine, Tohoku University, 2-1 Seiryo-machi, Aoba-ku, Sendai 980-8575, Japan; 2 National Cancer Center Hospital East, 6-5-1 Kashiwanoha, Kashiwa 277-8577, Japan; 3 Medical Corporation Kanno-Aiseikai Midorigaoka Hospital, 1-16 Nishitamagawa-cho, Shiogama 985-0045, Japan

**Keywords:** nurse, work values, latent profile analysis, human resources, hospitals, recruit, nursing students, nursing management

## Abstract

This study aimed to classify nurses with similar work values into subgroups by examining their intrinsic, extrinsic, social, and prestige work values. Additionally, we clarified the characteristics of the obtained subgroups using personal attributes, work engagement, and life satisfaction. Using a cross-sectional observational study design, we randomly sampled 52 hospitals in the Tohoku region of Japan and conducted a self-administered questionnaire survey with 2600 nurses. Latent profile analysis was performed to identify the number of subgroups. Of the 1627 collected questionnaires, 1587 were regarded as valid. The latent profile analysis revealed the following five subgroups with strong statistical significance: (1) self-oriented, (2) low, (3) medium-low, (4) medium-high, and (5) high types. The means of work engagement and life satisfaction gradually increased from the (2) low- to (5) high-type subgroups. There were significant differences among the subgroups in terms of marital status, child status, and job title. The (5) high-type subgroup had many nurses with job titles, high work engagement, and high life satisfaction. The (2) low-type subgroup included many nurses who were young, had few years of experience, were married, had children, and had low levels of work engagement and life satisfaction. Preregistration: This study was not registered.

## 1. Introduction

Women dominate the nursing profession worldwide. Although the male-to-female ratio varies slightly between countries, approximately 90% of nurses worldwide are female [1]. The social status of nurses also varies among countries; however, many countries have relatively high salaries for female nurses [2], and many women in these countries choose nursing as a stable and secure career [3]. The career development theory shows that individual work values play an important role in autonomous career choices for pursuing psychological success [4]. Due to the chronic shortage of nurses worldwide [5], it is important to clarify the work values emphasized by nurses, many of whom are women.

Nurses’ work values refer to their enduring beliefs about the positive conditions and outcomes they want to achieve through their work, as well as the principles and standards they value and that guide their attitudes, judgments, and actions about their work [6]. Work values consist of four subdomains—intrinsic (values related to work autonomy and growth), extrinsic (values related to job security and income), social (values related to contributing to society), and prestige (values related to authority and influence) [6]. Studies have examined nurses’ work values for securing human resources and providing safe and high-quality nursing care, along with their impact on patient-safety-culture attitudes [7] and organizational commitment [8]. However, to date, these studies have mainly focused on clarifying the relationship between nurses’ work values and specific outcomes. Although some studies have clarified what nursing students value [9], little is known about the kind of work values collectively held by nurses.

The key to understanding nurses’ work values is adopting a person-centered approach. A person-centered approach uses the quantity (from low to high levels) and quality of certain variables (e.g., the work value subdomains—intrinsic, extrinsic, social, and prestige) to identify constituent subgroups [10]. In other words, in terms of work values, how the four subdomains coexist within an individual is analyzed, and a subgroup of nurses with similar tendencies is obtained. Clarifying the nurse subgroups helps individual nurses recognize not only their own work values but also those of other nurses. Moreover, knowledge of the subgroups of nurses’ work values will enable nurse managers to understand the work values that nurses consider important and provide guidance and feedback that is tailored to nurses’ needs and motivations. Recent studies that adopted a person-centered approach to work values included a longitudinal sample of young people transitioning from students to workers in Finland [11], a sample of American lawyers [12], and a sample of Canadian agency workers [10]; the number of resulting subgroups was four, five, and five, respectively, of which the latter two worker samples contained level-specific profiles for the subdomains of work values, such as a subgroup with all subdomains being high, a subgroup with all subdomains being low, and so on. Each sample was also characterized by subgroups with particularly high levels of one or two subdomains. In contrast, the subgroups differed in the high and low subdomains among the young Finnish samples [11]. As the work values in the young Finnish samples’ study were measured before the participants chose a career or entered the workforce, they may have been categorized as diverse values.

In this study, work values were measured among nurses working in Japanese hospitals. Based on the previous research on choosing nursing as a secure career [3], we predicted that there would be a subgroup that emphasizes extrinsic work values (Hypothesis 1). Additionally, some nurses aim to become certified nurse specialists, nurse practitioners, and certified nurse administrators [13]. Therefore, we predicted that there would be subgroups that emphasize intrinsic and prestigious work values and level-specific profiles of work value subdomains, as demonstrated in studies using samples of American lawyers [12] and Canadian Government agency workers [10] (Hypothesis 2).

**Hypothesis** **1.** *One of the subgroups emphasizes extrinsic work values.*

**Hypothesis** **2.** *One of the subgroups emphasizes intrinsic and prestige work values.*

Furthermore, based on the obtained subgroups, we clarified the characteristics of each subgroup using individual variables, such as age, sex, number of children, and position as a nurse. A prior study using a sample of lawyers found sex differences between subgroups (e.g., women’s profiles were more likely to be characterized by stronger social work values than extrinsic and prestige work values) [12]. However, as approximately 90% of the participants in this study were female, these sex differences were not expected. Other personal attributes were analyzed exploratively without hypotheses as they have not been investigated in previous studies.

Based on the self-determination theory (SDT) [14], we examined how work engagement and life satisfaction scores differed between the subgroups. Work engagement is defined as “a positive and fulfilling work-related state of mind characterized by vitality, enthusiasm, and immersion” [15] (p. 187). Life satisfaction is defined as a person’s cognitive and affective evaluations of his or her life [16] (p. 187), and these are treated as positive outcomes. In SDT, a state of intrinsic job motivation has been shown to be associated with positive outcomes [14]. Specifically, regarding nurses’ work values, intrinsic work values have been demonstrated to have a significant positive impact on work engagement [17]. Therefore, we predicted that the subgroup that emphasized intrinsic work values would have higher work engagement. Similar to work engagement, based on SDT, we predicted that the group with higher intrinsic work values would also have higher life satisfaction (Hypothesis 3). However, nurses who did not value any of the work value subdomains did not value the work itself. Thus, if nurses valued things other than work and were satisfied with their lives, their life satisfaction scores were expected to be high, even if all work value subdomains were low (Hypothesis 4).

**Hypothesis** **3.** *Subgroups with greater intrinsic work values have higher levels of work engagement and life satisfaction.*

**Hypothesis** **4.** *When nurses value things other than work and are satisfied with their lives, their life satisfaction scores are high.*

Therefore, in this study, we aimed to classify nurses with similar value tendencies into subgroups using their intrinsic, extrinsic, social, and prestige work values. Furthermore, we clarified the characteristics of the obtained subgroups using personal attributes, work engagement, and life satisfaction. This study’s findings will facilitate not only nurses’ knowledge of the subgroup to which they belong but also allow them to refer to trends, such as those related to their personal attributes and work engagement levels within their subgroup. Nurse managers’ knowledge of which subgroups their nurses belong to will enable them to adopt a more appropriate approach to meet the work values of nurses.

## 2. Materials and Methods

This study adopted a cross-sectional observational design and complies with the STROBE checklist of items that should be included in published reports of cross-sectional studies [18].

### 2.1. Participants and Procedures

This study was conducted as part of a project to elucidate the work values and related factors of hospital nurses. Participants were recruited from hospitals with more than 100 beds in the Tohoku region of Japan. Although this study acquired data from October to December 2020, it did not include questions related to COVID-19 and its impact. Infections in the Tohoku region of Japan had been fewer than those in the Kanto region, which includes Tokyo. However, we selected only 50 nurses from each hospital to avoid burdening the hospitals. The sample size calculated and required for the project was 2600. Sample sizes were calculated using unpaired *t*-tests, assuming that the variables used in the study were compared between any two groups. Using the power analysis software G*Power 3.1.9.4, with an alpha error of 0.05, a power of 0.95, and an effect size of 0.2, the total sample size was 1302. If the collection rate by mail was 50%, the required number of samples was 2604. Therefore, for rounding purposes, we set the number of samples collected by the project at 2600. After calculating the sample size, 52 hospitals with 100 or more beds located in the Tohoku region of Japan were randomly sampled to recruit 2600 nurses. The researchers sent a set of 50 documents entirely in Japanese (including project and questionnaire explanations, questionnaires, and response envelopes) to the hospitals after obtaining permission from the nursing director. The nursing director selected 50 target nurses and provided them with the documents. The nurses responded to the questionnaire after reading the explanations and agreeing to participate. The completed questionnaires were placed in an anonymized envelope and deposited in a mailbox. The survey included registered and licensed nurses working in each hospital. They also included full-time and part-time employees. Nurses taking maternity, parental, or sick leave were excluded from the study.

A total of 2600 questionnaires were distributed, and 1627 were returned by mail. Of these, 1587 were considered valid responses (effective response rate of 61.0%), and 40 were excluded for being largely unanswered. The average age of the subjects was 40.7 years (±10.3), and 1464 (92.2%) were female. Table 1 lists the participants’ personal attributes.

### 2.2. Measures

#### 2.2.1. Demographics

We asked each participant about their demographic characteristics, including age, sex, years of experience as a nurse, position, educational background, marital status, and number of children.

#### 2.2.2. Nurses’ Work Values

The Nurses’ Work Values Scale [19] was used to assess nurses’ work values. This scale was developed in Japanese. For the four factors, the thirty-item scale included nine items for intrinsic work values (e.g., values for work autonomy and growth), five items for extrinsic work values (e.g., values for job security and income), ten items for social work values (e.g., values for contributing to society), and six items for prestige work values (e.g., values for authority and influence). Example items are “Improving nursing practice ability” and “Contributing to society by working as a nurse”. Items in this scale were answered on a five-point Likert scale ranging from 1 to 5 (1 = not at all important; 2 = not very important; 3 = slightly important; 4 = quite important; 5 = very important); the higher the score, the greater the importance of the work value for the nurse. Descriptive statistics and Cronbach’s α coefficients for all measures in this study are shown in Table 2.

#### 2.2.3. Work Engagement

The Japanese version of the Utrecht Work Engagement Scale (UWES) was used to assess work engagement [20]. The original scale developed by Schaufeli [21] has a three-factor structure—vigor, enthusiasm, and immersion. However, Shimazu et al. [20] clarified that the one-factor structure fits well; therefore, the analysis was performed with a nine-item one-factor structure in this study. Example items include “I am enthusiastic about my work” and “I feel proud of my work”. The participants responded on a seven-point Likert scale ranging from 0 (never) to 6 (always). The higher the score, the more the participant perceives themselves as engaged in their work. Descriptive statistics and Cronbach’s α coefficients for all measures in this study are shown in Table 2.

#### 2.2.4. Satisfaction with Life

For assessing life satisfaction, we used the Satisfaction with Life Scale (SWLS) developed by Diener et al. [22] and translated into Japanese by Oishi [23]. The SWLS is a five-item scale that includes items such as “In most respects, my life is close to my ideals” and “I am satisfied with my life”. Items were rated on a seven-point Likert scale from 1 (not applicable at all) to 7 (very applicable). The higher the score, the greater the satisfaction with life. Descriptive statistics and Cronbach’s α coefficients for all measures in this study are shown in Table 2.

### 2.3. Data Analysis

Data were analyzed using IBM SPSS version 26.0 for Windows (IBM Corporation, Armonk, NY, USA) and Mplus version 8.8 (Muthén and Muthén, 1998–2022). SPSS software was used for descriptive statistics and internal consistency calculations at the beginning of the analysis.

Mplus software was used to perform a latent profile analysis (LPA) to identify the optimal number of participant subgroups (latent profiles) based on the four subdomains measured by the nurses’ work values scale. As the number of items varied across the four subdomains of the nurses’ work values scale, we tested the validity of two to six subgroup models using standardized subdomain total scores. The best model was selected synthetically using theoretical support and conceptual interpretability of the profile along with the following statistical measures: Akaike information criterion (AIC), Bayesian information criterion (BIC), sample size adjusted BIC (SABIC), and bootstrap likelihood ratio test (BLRT).

BLRT was used to compare the statistical significance of the estimated models. This estimates the *p*-value using the 2 log likelihood difference and determines whether a particular model with k subgroups should reject a model with k − 1 subgroups [24]. For AIC, BIC, and SABIC, lower values indicate better model fit [24]. Prior literature shows that BLRT outperforms other information criteria, followed by BIC and SABIC [24]. Additionally, we examined the entropy value, which assesses the accuracy with which a model classifies individuals into the most probable subgroups. The entropy ranges from 0 to 1, with higher values indicating fewer classification errors [25].

After identifying the optimal number of subgroups, the nurses’ personal attributes, work engagement, and life satisfaction scores were used to test for mean-level differences. For this test, we used automated versions of the BCH [26] and DCAT methods [27] implemented in Mplus. These methods are preferred for analyzing distal outcomes, which are continuous and categorical variables across subgroups, as they allow for comparisons to be conducted between the subgroups while considering the attributes of the participants within the subgroups [26]. The BCH method was adopted to test for significant mean differences in age, years of experience, work engagement, and life satisfaction. The DCAT was applied to sex, marital status, child status, educational background, and job title.

### 2.4. Ethics Statement

The ethics committee of the institution with which the researcher is affiliated approved the study protocol. Hospital investigators and participants were assured confidentiality and anonymity during the research and publication processes. Before participation, we informed the participants about the purpose and design of the study and mentioned that participation was voluntary. The return of the questionnaire was deemed as consent for participation, and the study procedures conformed to the Declaration of Helsinki.

## 3. Results

The conformance indices are listed in Table 3. The AIC and ABIC decreased with an increasing number of subgroups, and the BIC was the lowest among the five subgroups. In addition, entropy was the highest among the five subgroups. As the BLRT showed non-significant results for the six subgroup solutions, the five solutions were determined to be the best models.

**Figure 1:**
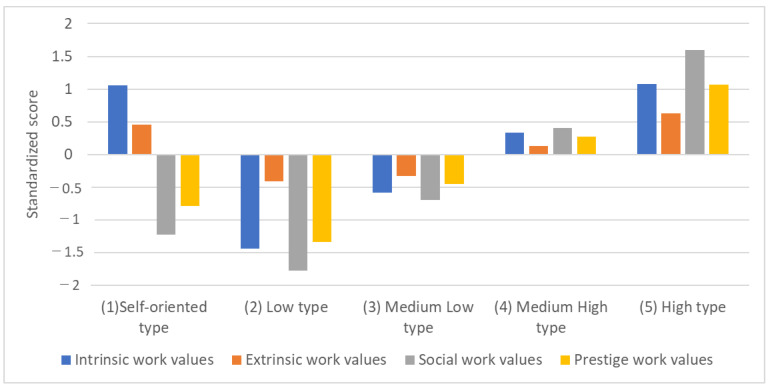
Distribution of work values in the five clusters.

The five subgroups obtained were named as follows (Figure 1): (1) self-oriented type with high intrinsic and extrinsic work values, (2) low type with the lowest work values, (3) medium-low type with slightly lower work values, (4) medium-high type with slightly higher work values, and (5) high type with high work values. Table 4 shows the number and probability of nurses belonging to each subgroup, and Table 5 compares the nurses’ work values in the four subdomains for each subgroup.

Table 6 and Table 7 compare the mean age, years of nursing experience, work engagement, and life satisfaction by subgroup using the BCH method. The (2) low-type subgroup was significantly younger than the (3) medium-low-, (4) medium-high-, and (5) high-type subgroups. Regarding years of experience, the (2) low-type subgroup had significantly less experience than the (4) medium-high-type and (5) high-type subgroups. Regarding work engagement and life satisfaction, the average values gradually increased significantly from the (2) low type to the (5) high type. The (1) self-oriented type was included in a specific subgroup.

Table 8 and Table 9 show the comparisons of sex, marital status, child status, job title, and educational background by subgroup using the DCAT method. Sex and educational background were not included in Table 8 and Table 9 because there were no significant differences between the subgroups. Regarding marital status, the (2) low-type subgroup had significantly more married people than the (4) medium-high-type and (5) high-type subgroups. Regarding parental status, the (2) low type was a significantly higher subgroup with one or more children than the (5) high type. Regarding job titles, the (4) medium-high-type and (5) high-type subgroups had significantly more nurses with job titles than did the (1) self-oriented-type, (2) low-type, and (3) medium-low-type subgroups. Furthermore, the (1) self-oriented-type subgroup had significantly fewer nurses with job titles than the (3) medium-low type. The (2) low type had an odds ratio of 1.94 for those who were married (2.5% CI:1.129, 97.5% CI:3.338) and 1.858 for nurses with at least one child (2.5% CI:1.088, 97.5% CI:3.173). No other subgroups or categories showed significant odds ratios.

## 4. Discussion

We conducted the present study with a sample of nurses working in Japanese hospitals to achieve the following two objectives: (1) to classify nurses with similar value tendencies into subgroups using the intrinsic, extrinsic, social, and prestige work values of nurses; and (2) to clarify the characteristics of the subgroups using personal attributes, work engagement, and life satisfaction. The results for the first objective showed statistically significant differences between the five subgroups. This was similar to the number of subgroups obtained in recent studies using LPA in a Finnish student-to-worker longitudinal sample [11], American lawyer sample [12], and Canadian Government worker sample [10]. These studies’ results supported the validity of the subgroup classification. Additionally, the latter two worker samples contained level-specific profiles for the subdomains of work values. In the present study, similar subgroups were obtained using a sample of nurses working in hospitals, thus supporting the results of previous studies. The number of subgroups and classification of the overall value tendencies showed trends similar to those of other worker subgroups. However, according to the results for the second objective, trends in the detailed work values and comparisons of personal attributes, means of work engagement, and life satisfaction yielded results specific to nurses. This was a newly elucidated finding of the present study. Specifically, three characteristics that were unique to nurses were revealed: (1) nurses comprised a group that attached the same importance to extrinsic work values; (2) many of the nurses classified into subgroups that emphasized the four subdomains of work values perceived themselves to hold positions of leaders and head nurses and had high levels of work engagement and life satisfaction; and (3) the subgroup that was low for all four subdomains of work values was younger, less experienced, and statistically more likely to include nurses who were married or had one or more children, and these nurses were also more likely to have low work engagement and life satisfaction. This supports the previous research [28] that work values differ by occupation. As this study elucidated the trends in nurses’ work values differentiated by subgroup, hospital nursing administrators should, thus, consider strategies tailored to each nurse’s work values to maintain and improve their motivation.

Regarding the average difference in work values among the subgroups, we hypothesized that one of the subgroups will emphasize extrinsic work values (Hypothesis 1), based on a previous study in which nurses were selected for having a stable and secure occupation [3]. However, in the present study, no significant differences were found in the means of extrinsic work values between the (2) low-type and (3) medium-low-type subgroups and between the (1) self-oriented-type and (5) high-type subgroups. Moreover, there were significant differences only between (2) and (4), (3) and (4), (4) and (1), and (4) and (5). Therefore, extrinsic work values generally hold similar levels of importance for nurses working in hospitals, compared to the other three work values, which differed significantly between the subgroups. In other words, among nurses working in Japanese hospitals, there is no subgroup that selects nursing as a particularly stable occupation; rather, they collectively place importance on the work values to the same extent. This finding supports the previous research that high school and college students who choose a nursing career retain some degree of their extrinsic work values [9].

Furthermore, because some nurses aim to become certified nurse specialists, nurse practitioners, and certified nurse administrators [13], we predicted that one of the subgroups will emphasize the intrinsic and prestige work values (Hypothesis 2). Profiles of the four subdomains of work values from the (2) low type to (5) high type were obtained by the level of work values. The (5) high type emphasized the most intrinsic and prestige work values, thus supporting the hypothesis. Furthermore, the (4) medium-high-type and (5) high-type subgroups had significantly more nurses in these positions than the (1) self-oriented-type, (2) low-type, and (3) medium-low-type subgroups, thereby supporting our hypothesis. Moreover, the (5) high type—the subgroup with the highest scores in all four subdomains containing intrinsic work values—had the highest levels of work engagement and life satisfaction. Based on SDT [14], subgroups that emphasized intrinsic work values were expected to have higher levels of work engagement and life satisfaction (Hypothesis 3), and this hypothesis was supported. Nurse managers can possibly increase the work engagement and life satisfaction of nurses by supporting them in obtaining more specialized qualifications, recommending training for becoming nurse managers, and respecting the will of the nurses.

In contrast, the (2) low-type subgroup was younger than the (4) medium-high and (5) high types, with fewer years of experience. In addition, the (2) low-type subgroup had a statistically high probability of including nurses who are married or have one or more children as well as lower work engagement and life satisfaction than the other three subgroups, excluding the (1) self-oriented type. This unexpected result suggests that young and inexperienced nurses working in Japanese hospitals are likely to have lower work engagement and life satisfaction after marriage and having children. Nurses who did not value any of the subdomains of work values—(2) low-type nurses—were considered to not place importance on the work itself. Therefore, it was hypothesized that if a nurse places importance on things other than work and is satisfied with their life, the score for life satisfaction will be high (Hypothesis 4). However, this hypothesis was not supported. Marriage and childbearing at a young age with low nursing experience may result in career disruptions during professional growth [29]. This can lead to reduced motivation, work values, work engagement, and life satisfaction. However, as this was a cross-sectional study, it is possible that nurses with low work values were more likely to be married and have children. Therefore, in the future, it will be necessary to longitudinally clarify the changes in work values occurring due to life events in hospital nurses. If marriage and childbearing at a young age, with little experience as a nurse, lead to a decline in work values, appropriate support from nurse administrators is necessary. The previous research has shown that supportive work environments and value alignment can prevent early turnover [30]. By listening to their thoughts and respecting their working preferences, nurse managers may be able to prevent young and inexperienced nurses from having their careers disrupted by marriage and childbearing while improving their work values, work engagement, and life satisfaction.

Finally, in the (1) self-oriented-type subgroup, intrinsic and extrinsic work values were at the same level as in the (5) high-type subgroup, but the social and prestige work values were at intermediate levels. In the (1) self-oriented-type subgroup, the number of nurses who perceived themselves as having a position was significantly lower than that in the (3) medium-low, (4) medium-high, and (5) high types, and work engagement was significantly higher than in the (2) low type and significantly lower than in the (4) medium-high and (5) high types. Therefore, it was predicted that (1) the self-oriented-type subgroup tended to work autonomously, although they did not aim for career advancement. Nurse administrators may be able to increase their work engagement by supporting the autonomy they aspire to have.

### 4.1. Implications for Nurses and Nursing Administrators

By comparing the nurses’ work values obtained in the results of this study, we were able to infer which subgroup the nurses belonged to. Nurses’ awareness of their work values and knowledge of which subgroup they belong to has the following benefit: by referring to the trends of the subgroup to which they belong, nurses can consider whether they can increase their work engagement by aiming for a promotion or by choosing a workplace that offers childcare support. This will enable nurses to choose a department or workplace that is suitable for them and help them consider how to improve their work engagement and life satisfaction.

For nurse managers, knowledge of which subgroups their nurses belong to allows them to respect the nurses’ intentions and work values and provide appropriate support. Such support from nurse administrators will help increase nurses’ work engagement and life satisfaction.

### 4.2. Limitations

As the participants in this study were hospital nurses from the Tohoku region of Japan, the results should be generalized with caution. Specifically, the Japanese Nursing Association reports that 92% of Japanese nurses are female [31], which is similar to the male-to-female ratio found in this study. The WHO statistics from 132 countries indicate that 89% of all nurses are female, and this proportion is the highest in the Western Pacific Region (95%) and lowest in Africa (76%) [1]. As the male-to-female ratio of nurses varies across countries, caution should be exercised when applying these findings to nurses working in countries outside Japan. Additionally, regarding the educational backgrounds of Japanese nurses, the number of nurses with a bachelor’s degree or higher is gradually increasing; even among new nurses, approximately 40% of them are graduates with a bachelor’s degree or higher, and most of them have an associate degree [31]. Among the participants of the present study, only 9.5% had a bachelor’s degree or higher, which is common in Japan. Similar to the male-to-female ratio of nurses, the educational backgrounds of nurses also vary by country, and hence, caution should be exercised in generalizing our results to nurses working in other countries. In the future, research targeting nurses working in various regions and facilities will be beneficial for nurse managers and human resource management.

Regarding the data acquisition method used in this study, 50 nurses were selected by nurse administrators from each hospital; however, there is a possibility of selection bias, such as due to the ease of asking nurses to participate. In addition, because a self-administered questionnaire was used, it is possible that the participants provided socially desirable answers or were biased by overestimating or underestimating themselves. Although certain measures, such as using scales whose reliability and validity have been examined scientifically, were taken in this study, it is difficult to completely eliminate these biases.

In addition, because this was a cross-sectional study, the changes in participants over time and individual differences could not be clarified, and the causal relationships between the variables could not be strictly described. Therefore, in future research, it will be necessary to longitudinally clarify the changes in work values due to the life events of hospital nurses. Such longitudinal studies will reveal whether nurses who do not place importance on any of the four work value subdomains have higher rates of marriage and childbearing or whether work values are altered by marriage and childbearing.

Finally, the COVID-19 pandemic may have affected our findings. The previous research indicated that extrinsic values tended to temporarily increase after the 9/11 terrorist attacks [32] and war [33]. Although the present study may have demonstrated similar results, the impact of COVID-19 could not be determined due to the cross-sectional study design. In the future, it is necessary to verify the impact of COVID-19 on nurses’ work values, such as through a longitudinal study.

## 5. Conclusions

Using latent profile analysis on four subdomains of nurses’ work values, the following five subgroups were obtained: (1) self-oriented type, (2) low type, (3) medium-low type, (4) medium-high type, and (5) high type. The (5) high-type subgroup had many nurses with job titles and high levels of work engagement and life satisfaction. In contrast, the (2) low type included many young nurses who had a few years of experience, were married, had children, and had low levels of work engagement and life satisfaction. These findings suggest that young and inexperienced nurses working in Japanese hospitals may exhibit lower levels of work engagement and life satisfaction after getting married and having children.

## Figures and Tables

**Table 1:** Participants’ basic attributes (N = 1587).

	n (%)	Mean (SD)
Sex		
Female	1464 (92.2)	
Male	123 (7.8)	
Mean age (SD) in years		40.7 (10.3)
Mean years of nursing experience (SD)		17.9 (10.1)
Marital status		
Married	1013 (63.9)	
Single	529 (33.4)	
Other (divorce, bereavement, etc.)	44 (2.8)	
Number of children		
1 or more	955 (60.3)	
None	630 (39.7)	
Educational background		
Baccalaureate program (4-year program in nursing) or master’s program in nursing	150 (9.5)	
Vocational school or junior college for registered nurses	1435 (90.5)	
Position		
Director, Deputy Director, Head Nurse, Assistant Head Nurse, Team Leader	504 (31.8)	
Regular nurse	1083 (68.2)	

Note: SD: standard deviation.

**Table 2:** Descriptive statistics of the scales and Cronbach’s α coefficients (N = 1587).

Scale	Number of Items	Range	Mean	SD	Cronbach’s α
Nurses’ work values scale					
Intrinsic work values	9	15–45	35.2	5.7	0.92
Extrinsic work values	5	7–25	18.6	4.1	0.83
Social work values	10	10–50	36.3	7.2	0.94
Prestige work values	6	6–30	16.8	5.3	0.92
Utrecht Work Engagement Scale	9	0–54	22.4	9.1	0.93
Satisfaction With Life Scale	5	5–35	20.3	5.6	0.90

Note: SD: standard deviation.

**Table 3:** Results from latent profile analyses (N = 1587).

Subgroup	AIC	BIC	ABIC	Entropy	BLRT
2	16,795.750	16,865.555	16,824.256	0.726	<0.001
3	16,495.727	16,592.380	16,535.198	0.730	<0.001
4	16,479.869	16,603.370	16,530.304	0.791	<0.001
5	16,295.646	16,445.995	16,357.045	0.844	<0.001
6	16,278.295	16,455.492	16,350.658	0.761	1.000

Note: AIC: Akaike information criteria; BIC: Bayesian information criteria; ABIC: sample-size-adjusted BIC; BLRT: bootstrap likelihood ratio test.

**Table 4:** Number of people in each subgroup and probability of belonging.

Subgroup	Number of People (%)	Probability of Belonging				
		1	2	3	4	5
1	18 (1.1)	0.81	0.02	0.15	0.03	0.00
2	100 (6.3)	0.01	0.86	0.13	0.00	0.00
3	604 (38.1)	0.01	0.03	0.89	0.07	0.00
4	641 (40.4)	0.00	0.00	0.05	0.91	0.03
5	224 (14.1)	0.00	0.00	0.00	0.09	0.91

**Table 5:** Comparison of the means of work values by subgroup.

	(1) Self-Oriented		(2) Low		(3) Medium-Low		(4) Medium-High		(5) High		
	M	SE	M	SE	M	SE	M	SE	M	SE	
Intrinsic work values	1.06	0.38	−1.44	0.14	−0.58	0.11	0.34	0.04	1.08	0.05	(2) < (3) < (4) < (1), (5)
Extrinsic work values	0.46	1.18	−0.41	0.14	−0.33	0.06	0.13	0.04	0.63	0.07	(2), (3) < (4) < (1), (5)
Social work values	−1.23	0.67	−1.78	0.10	−0.70	0.03	0.40	0.04	1.60	0.04	(2) < (1) < (3) < (4) < (5)
Prestige work values	−0.78	1.15	−1.34	0.09	−0.45	0.06	0.28	0.04	1.06	0.07	(2) < (1), (3) < (4) < (5)

Note: If there is a significant difference in the mean, it is described as <. M: mean; SE: standard error.

**Table 6:** Comparison of the means and standard errors of covariates by subgroup.

	(1) Self-Oriented		(2) Low		(3) Medium-Low		(4) Medium-High		(5) High	
	M	SE	M	SE	M	SE	M	SE	M	SE
1. Age	37.10	2.48	37.81	1.08	40.87	0.47	40.89	0.46	41.45	0.81
2. Years of nursing experience	14.62	2.20	15.51	1.10	17.95	0.46	18.08	0.45	18.61	0.79
3. Work engagement	17.02	2.93	10.29	0.93	19.10	0.36	25.11	0.34	29.17	0.66
4. Life satisfaction	18.65	2.22	16.44	0.71	19.29	0.24	21.06	0.24	22.37	0.43

Note: M: mean; SE: standard error.

**Table 7:** Comparison of the means and standard errors of covariates by subgroup.

	(1) vs. (2)		(1) vs. (3)		(1) vs. (4)		(1) vs. (5)		(2) vs. (3)		(2) vs. (4)		(2) vs. (5)		(3) vs. (4)		(3) vs. (5)		(4) vs. (5)	
	χ2	*p*	χ2	*p*	χ2	*p*	χ2	*p*	χ2	*p*	χ2	*p*	χ2	*p*	χ2	*p*	χ2	*p*	χ2	*p*
1.	0.07		2.14		2.25		2.77		6.15	*	6.95	**	7.27	**	0.00		0.38		0.33	
2.	0.13		2.12		2.38		2.93		3.86		4.73	*	5.28	*	0.03		0.53		0.31	
3.	4.70	**	0.48		7.54	**	16.39	***	72.85	***	226.94	***	275.46	***	131.91	***	181.69	***	27.25	***
4.	0.88		0.08		1.16		2.70		13.34	***	37.93	***	50.98	***	23.80	***	40.04	***	6.55	*

Note: *** *p* < 0.001; ** *p* < 0.01; * *p* < 0.05.

**Table 8:** Comparison using categorical outcomes by subgroup.

	(1) Self-Oriented		(2) Low		(3) Medium-Low		(4) Medium-High		(5) High	
	Prob	SE	Prob	SE	Prob	SE	Prob	SE	Prob	SE
1. Marital status (married)	0.29	0.15	0.46	0.06	0.34	0.02	0.32	0.02	0.30	0.03
2. Number of children (1 or more)	0.34	0.17	0.51	0.06	0.39	0.02	0.40	0.02	0.36	0.03
3. Position (Director, Deputy Director, Head Nurse, Assistant Head Nurse, Team leader)	0.09	0.10	0.19	0.05	0.28	0.02	0.35	0.02	0.41	0.04

Note: Prob: probability; SE: standard error.

**Table 9:** Comparison using categorical outcomes by subgroup.

	(1) vs. (2)		(1) vs. (3)		(1) vs. (4)		(1) vs. (5)		(2) vs. (3)		(2) vs. (4)		(2) vs. (5)		(3) vs. (4)		(3) vs. (5)		(4) vs. (5)	
	χ2	*p*	χ2	*p*	χ2	*p*	χ2	*p*	χ2	*p*	χ2	*p*	χ2	*p*	χ2	*p*	χ2	*p*	χ2	*p*
1.	1.12		0.08		0.04		0.01		3.47		5.30	*	5.58	*	0.26		0.70		0.19	
2.	0.96		0.09		0.15		0.02		3.62		3.26		5.13	*	0.13		0.53		0.98	
3.	0.82		4.04	*	6.78	**	9.72	**	2.96		9.51	**	14.18	***	3.87	*	9.23	**	2.27	

Note: *** *p* < 0.001; ** *p* < 0.01; * *p* < 0.05.

## Data Availability

The datasets used in the current study are available from the corresponding author upon reasonable request.

## Public Involvement Statement

No public involvement in any aspect of this research.

## Guidelines and Standards Statement

This manuscript was drafted against the Strengthening reporting of observational studies in epidemiology (STROBE) Statement [18] for a cross-sectional observational research.
